# Development of a Predictive Model for N-Dealkylation of Amine Contaminants Based on Machine Learning Methods

**DOI:** 10.3390/toxics12120931

**Published:** 2024-12-22

**Authors:** Shiyang Cheng, Qihang Zhang, Hao Min, Wenhui Jiang, Jueting Liu, Chunsheng Liu, Zehua Wang

**Affiliations:** 1 School of Envronment and Spatial Informatics, China University of Mining and Technology, XuZhou 221116, China; csy1402@cumt.edu.cn (S.C.); ts23160081a31@cumt.edu.cn (Q.Z.); ts21160158p31@cumt.edu.cn (H.M.); 15660053610@163.com (W.J.); 2 School of Computer Science and Technology, China University of Mining and Technology, XuZhou 221116, China; 3 School of Environmental Studies, China University of Geosciences, Wuhan 430079, China; cliu@cug.edu.cn; 4 The Department of Electrical and Computer Engineering, University of British Columbia, Vancouver, BC V6T 1Z4, Canada; zwang@ece.ubc.ca

**Keywords:** machine learning, biotransformation, cytochrome P450 enzymes, amine contaminants, N-dealkylation reaction, binary classification

## Abstract

Amines are widespread environmental pollutants that may pose health risks. Specifically, the N-dealkylation of amines mediated by cytochrome P450 enzymes (P450) could influence their metabolic transformation safety. However, conventional experimental and computational chemistry methods make it difficult to conduct high-throughput screening of N-dealkylation of emerging amine contaminants. Machine learning has been widely used to identify sources of environmental pollutants and predict their toxicity. However, its application in screening critical biotransformation pathways for organic pollutants has been rarely reported. In this study, we first constructed a large dataset comprising 286 emerging amine pollutants through a thorough search of databases and literature. Then, we applied four machine learning methods—random forest, gradient boosting decision tree, extreme gradient boosting, and multi-layer perceptron—to develop binary classification models for N-dealkylation. These models were based on seven carefully selected molecular descriptors that represent reactivity-fit and structural-fit. Among the predictive models, the extreme gradient boosting shows the highest prediction accuracy of 81.0%. The SlogP_VSA2 descriptor is the primary factor influencing predictions of N-dealkylation metabolism. Then an ensemble model was generated that uses a consensus strategy to integrate three different algorithms, whose performance is generally better than any single algorithm, with an accuracy rate of 86.2%. Therefore, the classification model developed in this work can provide methodological support for the high-throughput screening of N-dealkylation of amine pollutants.

## 1. Introduction

**Figure 1:**
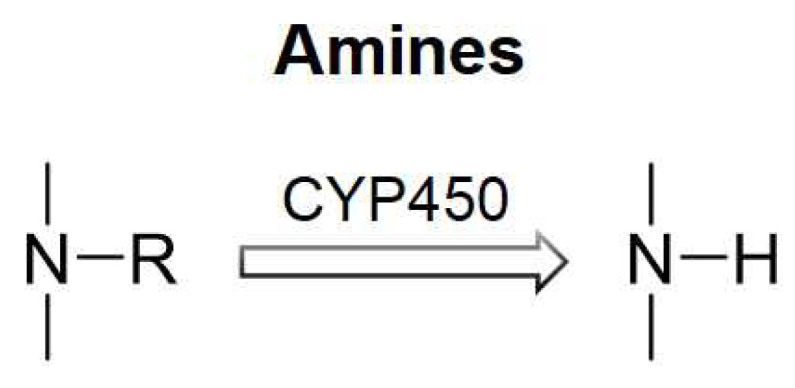
The N-dealkylation of amine contaminants mediated by cytochrome P450 enzymes.

In alkylated amines, the lone pair of electrons on the nitrogen atom increases their solubility, while substituents attached to the nitrogen increase their lipophilicity. This combination of properties is essential for the molecule’s ability to cross cellular membranes, which explains why alkylated amines are frequently included in pharmaceutical agents [1]. Additionally, these functional groups used extensively in pesticides, industrial additives, and personal care products, among others. Since they are often not chemically bound to the product matrix, alkylated amines has been reported to be common micropollutants in various environmental matrices worldwide [2]. They can accumulate in humans, with increasing evidence of health risks [3,4]. There are different types of alkylated amines. Aliphatic amines have straight or branched carbon chains, while aromatic amines contain aromatic rings. When metabolized by cytochrome P450 (CYP450) in organisms, alkylated amines can undergo cleavage of N-C bond, a process known as N-dealkylation. This reaction results in a lower-order amine metabolite ($R_{2}\mathrm{NH}$, as shown in Figure 1) and an aldehyde/ketone metabolite (RCOH/RCOR) [5]. N-dealkylation biotransformation mediated by CYP450 can significantly impact the clearance rates and toxicological properties of alkylated amine pollutants [6]. On the one hand, a subset of aldehyde and ketone metabolites produced during N-dealkylation, particularly those involving the alpha, beta-unsaturated carbonyls, can form stable adducts with proteins, glutathione, or DNA in living organisms. This reaction can potentially lead to various adverse outcome pathways, which begin with the fundamental electrophile–nucleophile reaction as the molecular initiating event [7,8,9,10,11]. On the other hand, it has also been established that the lower-order aromatic amines (AAs), resulting from N-dealkylation on the nitrogen side, are highly toxic to fish and mammals [12]. In addition to their toxicity, aromatic amines can decompose into other carcinogens through the process of N-hydroxylation as the molecular initiating event [13]. However, not all alkylated amines can undergo N-dealkylation. This limitation may arise because the metabolic regions of some alkylated amines cannot enter the active site of the CYP450, or it could be due to the substrate itself is nearly reactively inert [14]. Assessing the feasibility of N-dealkylation biotransformation of amine pollutants in humans is essential for evaluating the health risks associated with these pollutants.

The increasing number of emerging amine pollutants poses challenges for evaluating the feasibility of N-dealkylation metabolism, especially when using conventional mass spectrometry-based metabolite identification or quantum chemical calculations for mechanism analysis. Typically, high-sensitivity mass spectrometry is employed to identify N-dealkylation metabolites from samples extracted from human liver microsomes (HLMs) or the livers of mice exposed to alkylated amines. However, this method incurs significant time and material costs, is constrained by the “3R principle”, making it challenging to apply for the rapid screening of large quantities of amine pollutants through N-dealkylation metabolism [15,16]. On the other hand, quantum chemical calculations utilize computer simulations to investigate the biotransformation of amine pollutants mediated by CYP450. These simulations can reveal the electronic structure properties that influence transformation mechanisms, the thermodynamic characteristics of N-dealkylation metabolism, and the structures of the resulting metabolites. However, assessing reactivity through this method requires a strong understanding of theoretical computational chemistry and practical experience. Additionally, there are challenges such as locating transition states and intermediates, as well as the high computational resource demands of enzyme systems [17,18]. Consequently, applying this approach to high-throughput screening of N-dealkylation reactions is also difficult.

It is essential to develop a rapid method for assessing whether amines undergo N-dealkylation biotransformation mediated by CYP450. This is crucial for evaluating the environmental health risks of emerging amine pollutants. With the growing amount of experimental data and advancements in high-performance computing, various machine learning algorithms have shown strong classification performance and significant potential for applications in identifying sources of environmental pollutants and predicting their toxicity [19,20,21]. Machine learning methods that utilize descriptors to predict compound properties are straightforward, reliable, and do not necessitate extensive training. These algorithms are computationally cost-effective, enabling them to run efficiently on standard computer systems. However, unlike drug metabolism predictions, there are currently few machine learning models that take into account the key biotransformation pathways of pollutants. Additionally, there is no existing dataset specifically for CYP450-mediated biotransformation of amine pollutants (N-dealkylation). Furthermore, many machine learning algorithms are often criticized for their “black box” prediction process, which lacks sufficient interpretability [22].

**Figure 2:**
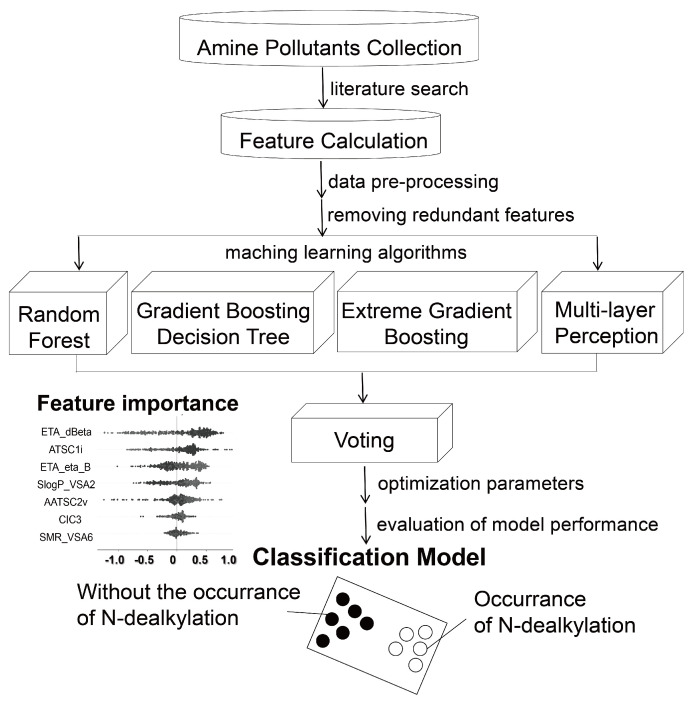
Scheme for building the model for predicting the occurrence of N-dealkylation reaction.

Therefore, this study systematically gathers data on the N-dealkylation of amine pollutants metabolized by P450 enzymes reported in the literature to curate a large dataset. Then optimal molecular descriptors were selected, the predictive performance of four classical machine learning algorithms was compared, and significant descriptors influencing N-dealkylation were identified. Ultimately, an effective predictive model for the N-dealkylation reaction of emerging amine pollutants was developed using a voting model. Figure 2 is illustrates the process of model structure in this work.

## 2. Materials and Methods

### 2.1. Dataset Establishment

To build a N-dealkylation prediction model for amine pollutants, an amine pollutant dataset is built that contains 286 amine pollutants. These pollutants are widely distributed in multiple application areas: food additives (9), pesticides (6), pharmaceuticals (130), personal care products (16), and other industrial products (125). These substances are classified as Chemicals of Emerging Concern (CECs) and are documented in HMDB v5.0 [23] as well as in the CECscreen data library [24] by labeling each amine pollutant sample in the dataset based on whether it has been reported to undergo an N-dealkylation reaction in metabolic experiments. If there exists such a metabolic reaction, label the sample as “yes”; otherwise, label the sample as “no”. Data were imported and analyzed with Python 3.8 using libraries such as Numpy, pandas, and Scipy. Then the amine pollutant dataset was split into the training set for model training and the testing set for model testing. The “train_test_split” function in scikit-learn is used for the selection of the training and test set. It randomly selects 80% of the dataset to serve as the training set, while the remaining 20% is used as the test set (test_size equal to 0.2). The proportions of yes and no labels remain consistent across the original dataset, training set, and test set. The training set is utilized to build and refine the model, while the test set, which is not involved in the training process, is employed to evaluate the model’s predictive performance on unseen data.

### 2.2. Feature Selection

Molecular descriptor screening is a key step in establishing a prediction model. In this work, the compound structure in the amine pollutant dataset is first converted into the SMILE strings (Simplified Molecular Input Line Entry System). SMILEs are then subject to feature extraction using the Python package called Mordred [25]. Mordred systematically calculates two-dimensional descriptors for the studied molecules, covering a total of 1613 default molecular descriptors that belong to 43 modules. All calculated descriptors are then filtered based on the following principles: Descriptors with more than one missing value, a high correlation to related descriptors (where the correlation coefficient exceeds 0.9), or too many duplicate values (with a variance less than 0.1) are excluded. Following the initial screening, the CorrelationAttributeEval method available in the Weka 3.8.6 machine learning platform is utilized to rank descriptors according to their classification power [26].

### 2.3. Machine Learning Algorithms Utilized

Four widely used machine learning classification algorithms, recognized for their robustness and stability, were employed to build the classifier. These algorithms include random forest (RF) [27], gradient boosting decision tree (GBDT) [28], extreme gradient boosting (XGB) [29], and multi-layer perceptron (MLP) [30]. In order to improve the robustness and accuracy of the model, the voting algorithm is utilized to integrate the single model and generate a hybrid prediction model [31]. The machine learning algorithms were were carried out using scikit-learn.

**Random forest.** The random forest algorithm is an ensemble method based on the decision tree that is widely used in classification and regression problems. The idea is to build multiple decision trees using a decision tree algorithm and ensure model diversity through random feature selection. Finally, the random forest integrates the prediction results of multiple decision trees through a voting mechanism

**Gradient boosting decision tree.** Gradient boosting decision tree is a machine learning algorithm that combines multiple ‘weak’ learning models to generate a single, more accurate “strong” model. The weak models are typically decision trees; in the training process, the weak new model is literately added to the strong model to reduce the loss function of the previous strong model. The final prediction result represents the sum of the single results of all the models.

**Extreme gradient boosting.** Extreme gradient boosting is also a boosting machine learning algorithm. Compared with the sequential learning in GBDT, XGB provides parallel tree boosting that significantly speeds up the boosted tree algorithm.

**Multi-layer perceptron.** Multi-layer perceptron is a kind of feedforward neural network consisting of an input layer, an output layer, one or more hidden layers, and an activation function. The MLP is widely used in various fields including image recognition, natural language processing, and data classification.

**Voting algorithm.** Soft voting is a strategy used in ensemble learning for classification problems. It operates on the principle that the minority should follow the majority, which helps reduce variance by combining multiple models to enhance the robustness of the overall model. This robustness refers to the algorithm’s ability to tolerate changes in the data. Soft voting assigns the final class label by weighing the predicted outcomes from various base classifiers. Ideally, the predictive performance of a voting method should exceed that of any single base model.

### 2.4. Evaluation of Model Performance

To evaluate the performance of the prediction model, a set of performance metrics have been applied including **accuracy, precision, sensitivity, specificity, F1 score**, area under the ROC curve (ROC), and the Matthews correlation coefficient (MCC).

Accuracy represents the overall correctness of all classes; it can be calculated as:

$$Accuracy=\frac{correct\;classifications}{total\;classifications}=\frac{TP+TN}{TP+TN+FP+FN}$$

Precision measures the quality of a positive prediction made by the model, it refers to the number of true positives divided by the total number of positive predictions:

$$Precision=\frac{TP}{TP+FP}$$

Sensitivity (recall) measures the proportion of actual positive cases classified by the prediction model, it is also called true positive rate (TPR), it mathematically can be defined as:

$$Sensitivity=\frac{correctly\;classified\;positives}{all\;acutal\;positives}=\frac{TP}{TP+FN}$$

Specificity measures the proportion of true negative samples which are correctly identified by the model, it can also be called the true negative rate (TNR).

$$Specificity=\frac{TN}{TN+FP}$$

F1 score is a measure of the harmonic mean of precision and sensitivity (recall), it symmetrically represents both precision and recall in one metric. The F1 score can be calculated by the following equation:

$$F_{1}=2\cdot \frac{Precision\cdot Recall}{Precision+Recall}=\frac{2TP}{2TP+FP+FN}$$

The ROC curve represents the receiver operating characteristic curve—it is a graphical representation that illustrates how well the model can separate the positive and negative classes. The x-axis of the ROC curve is 1-*specificity* (false positive rate), the y-axis is *sensitivity* (true positive rate). The range of ROC AUC score values from 0 to 1, where higher values indicate better performance.

Matthews correlation coefficient, also abbreviated as MCC, is a measure of the performance of binary classification. The MCC is in essence a correlation coefficient between the observed and predicted binary classifications, it can be calculated by the following formula:

$$MCC=\frac{TP\times NP-FP\times FN}{\sqrt{\left(TP+FP)(TP+FN)(TN+FP)(TN+FN\right)}}$$

where true positive (TP) means predicted positive samples that are actually positive, false positives (FP) means predicted positive samples that are actually negative, false negatives (FN) means predicted negative samples that are actually positive, true negatives (TN) means predicted negative samples that are actually negative. The MCC value of a classification model greater than 0.4 indicates qualified predictive ability; the higher the value, the better the model’s predictive performance [31]. Ultimately, the AMBIT Discover software was utilized to compute the Euclidean distances of the compounds within both the training and test sets, and the application domain of the prediction model based on molecular descriptors was assessed [32].

## 3. Results

### 3.1. Data Collection and Feature Selection

The dataset constructed in this work includes 155 amines that can undergo N-dealkylation mediated by human CYP450 enzymes, while 131 amines have not been reported to undergo N-dealkylation mediated by CYP450 in the human body. The 286 amines were used for feature extraction by Mordred, followed by the elimination of redundant features, as described in Section 2. This process reduced the number of descriptors from 1613 to 278. Additionally, by employing an attribute evaluator method, we identified the optimal feature subset containing seven descriptors that had the highest correlation with the classification target. These seven descriptors (Table 1) were then used to build the following models: random forest (RF), extreme gradient boosting (XGB), multi-layer perceptron (MLP), and gradient boosted decision trees (GBDT). The N-dealkylation reaction is a free radical reaction. The likelihood of this reaction occurring depends on the reactivity-fit between amine and the active species of CYP450, referred to as compound I (Cpd I). Additionally, the reaction’s occurrence is influenced by the structure-fit between the substrate and enzyme. The molecular properties represented by the seven selected descriptors are associated with both reactivity-fit and structural-fit [33].

Specifically, **ATSCli** and **AATSC2v** both belong to autocorrelation descriptors; they reveal the electronic distribution and volume structure inside the molecule from different perspectives. **ETA_eta_B** and **ETA_dBeta** belong to the extended topological chemical atomic descriptor and characterize the degree of molecular branching and local electronic environment differences from different perspectives. **CIC3** reflects the strength of interactions between charge centers within a molecule. **SMR_VSA6** and **SlogP_VSA2** belong to MoeType molecular descriptors, representing the character of molecular charge distribution and hydrophobicity [25].

### 3.2. Single Machine Learning Prediction Model

This work first builds four prediction models including a random forest, a gradient boosting decision tree, an extreme gradient-boosting model, and a multi-layer perceptron. The optimized hyperparameters for each prediction model are shown in the Table 2.

**Figure 3:**
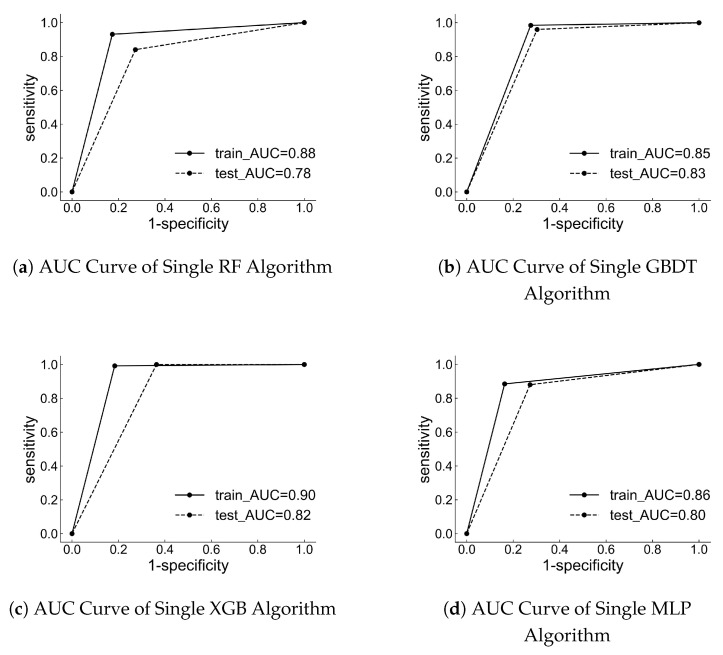
AUC curves of single machine learning classifier.

To assess the performance of the individual models, Table 3 and Table 4 present the accuracy, sensitivity, specificity, precision, F1 score, and Matthews correlation coefficient values for both the training and testing sets. Additionally, the results of the corresponding receiver operating characteristic curve and the area under the curve are displayed in Figure 3. The results indicate that the Matthews correlation coefficient values for the test sets of four classification models are all higher than 0.56. Furthermore, the corresponding receiver operating characteristic curves are located in the upper left corner, and the area under the curve is greater than 0.78. These findings suggest that the prediction results of these four classifiers have substantial predictive value. Among the four machine learning classifiers, the GBDT and XGB prediction model demonstrate superior performance. GBDT achieved an accuracy of 81.0%, while XGB reached 79.3%. Additionally, GBDT had a lower false negative rate of 4.0%, and XGB had an impressive false negative rate of 0.0%.

### 3.3. Model Explanation

**Figure 4:**
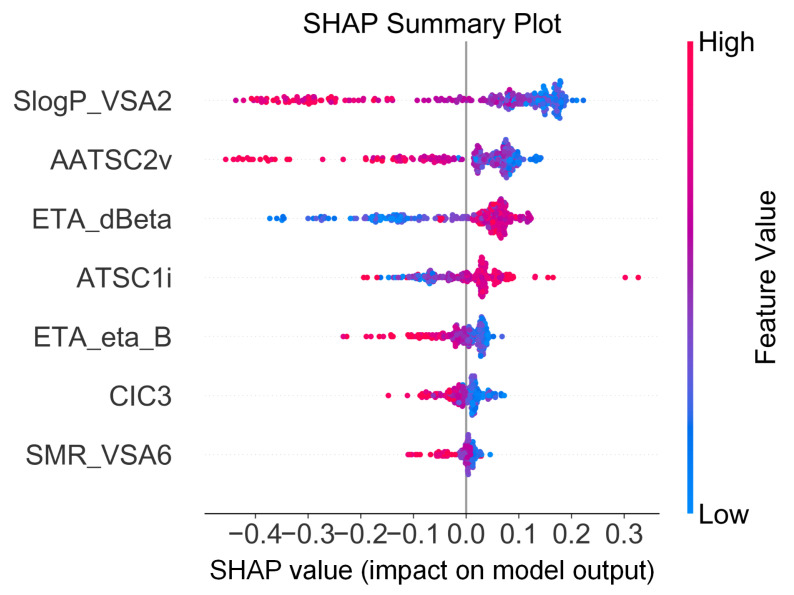
SHAP summary plot of features’ contribution.

The impact of the seven input descriptors on N-dealkylation feasibility predictions was evaluated using Shapley values. Shapley values quantify the contribution of each descriptor to the predicted results as illustrated in the hierarchy of the SHAP summary plot (Figure 4). In this figure, each row represents a descriptor, with the horizontal axis indicating the corresponding Shapley value. A positive Shapley value suggests that an increase in the input descriptor is linked to the output of the occurrence of N-dealkylation. In contrast, a negative Shapley value indicates that an increase in the input descriptor is associated with the output of the non-occurrence of the reaction. The SHAP summary plot ranks descriptors by the average absolute Shapley values, highlighting the most significant descriptors and vice versa [34].

Among the seven selected descriptors employed in prediction, the MoeType descriptor SlogP_VSA2 is the most significant descriptor affecting N-dealkylation prediction. Furthermore, AATSC2v, ETA_dBeta, and ATSCli also provide excellent classification ability. Thus, the following molecular information were important, namely:

- **SlogP_VSA2** defined using MoeType functions using electrostatic potential on the van der Waals surface of an organic spacer [25].
- **AATSC2V** defined using autocorrelation functions using averaged and centered Moreau–Broto autocorrelation of lag 2 weighted by vdw volume [25].
- **ETA_dBeta** defined using ETA functions using with the difference between contributions from sigma bonds and non-sigma bonds (pi-bonds) [25].
- **ATSC1i** defined using autocorrelation functions using averaged centered Broto–Moreau autocorrelation weighted by van der Waals volumes [35].

### 3.4. Voting Ensemble Learning (VEL) Approach for Final Predictions

**Figure 5:**
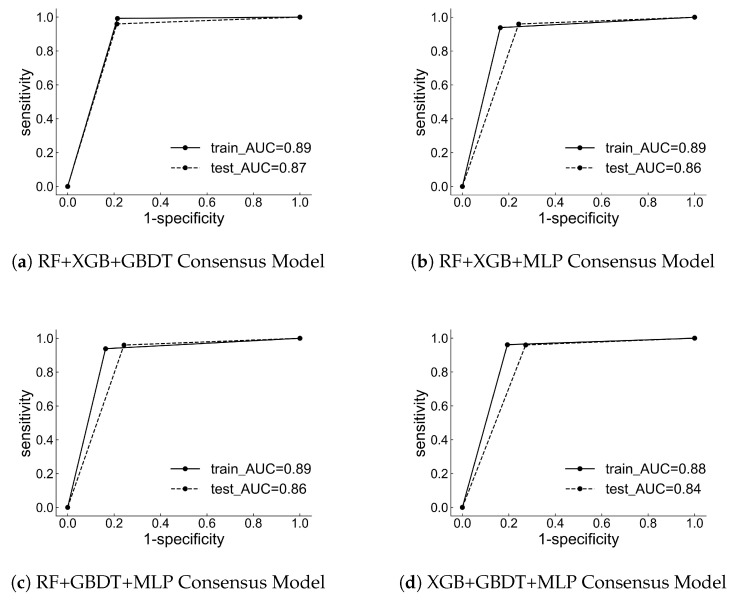
AUC curves of consensus machine learning classifier.

Since each machine learning model predicts a different set of compounds, this study employs an approach that integrates the results of these individual models to leverage their strengths and enhance overall predictive performance. The study combines the individual models in various configurations using a soft voting-based strategy. In this method, the final class label is determined by weighing the predicted outcomes according to the accuracy rate of each base classifier. Table 5 and Table 6 present the accuracy, sensitivity, specificity, precision, F1 score, and Matthews correlation coefficient values for both the training and testing sets of the different soft voting models. Additionally, the corresponding receiver operating characteristic curve and the area under the curve are displayed in Figure 5.

Combining three of the four prediction models in different ways significantly enhanced predictive performance over using each model individually. As a result, we chose the consensus models as our final prediction strategy. Among the different consensus models that utilized various combinations of base classifiers, the best performing model in our experiment combined the RF, XGB, and GBDT algorithms. This model achieved an accuracy of 84.20%, a false negative rate of 4%, and an MCC of 0.74.

### 3.5. Application Domain

The application domain (AD) of a model refers to the range of molecular descriptors of the amines included in the training set, which establishes the boundaries for the model’s applicability. If the molecular descriptor of the compound to be predicted falls within this AD range, the model can provide reliable predictions. However, if it falls outside this range, the model is not suitable for use. Table 1 displays the range of molecular descriptors for the model along with their mean values.

We utilize Euclidean distance to evaluate the suitability of compounds for the established model. The maximum Euclidean distance for the compound in training set is 1.35 (the cutoff value). The calculated Euclidean distances for all compounds in the test set are below this threshold, indicating that all compounds in the test set are appropriate for the prediction model. Furthermore, this criterion provides a basis for assessing whether compounds with unknown activity are suitable for the established model.

### 3.6. Misclassification Analysis

**Figure 6:**
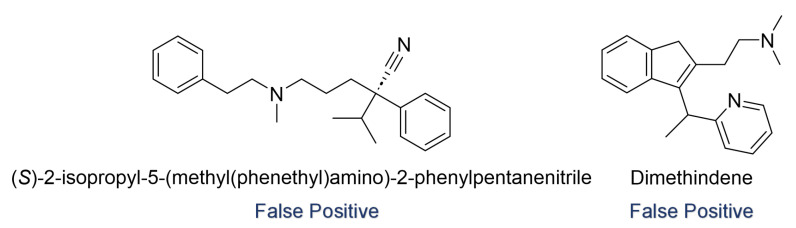
Amines that were misclassified in the test set.

We performed a misclassified analysis of the best consensus model results by categorizing the false positive (FP) and false negative (FN) errors based on our features. In the test set, the best consensus model misclassified a total of nine amines, resulting in eight false positives and one false negative. Among these, six amines had descriptor values that exceeded the mean ± standard deviation of the compounds in the training set. Figure 6 illustrates the characteristics of the remaining two amine compounds that were misclassified by the best consensus model, even though their descriptors fell within the mean ± standard deviation range. Both of these compounds are classed as false positives. This suggests that the model’s misclassifications are more likely to result in false positive errors.

## 4. Discussion

A large portion of organic pollutants have N-alkyl components that are often the first point of attack during environmental transformation by both abiotic and biotic processes [36,37]. Understanding the feasibility of N-dealkylation for emerging amine pollutants is essential for evaluating risks to human and environmental health. This study presents a predictive model for assessing the feasibility of N-dealkylation in these pollutants using machine learning methods for the first time. The findings demonstrate that machine learning techniques can be effectively employed in environmental research to identify key biotransformation pathways, specifically N-dealkylation metabolism, for amine pollutants. It is noted that the application of machine learning to identify the feasibility of key biotransformation routes for pollutants has been much less studied compared to drug metabolism [33]. These results suggest the potential for developing more comprehensive models that integrate metabolic routes and reactivity for emerging pollutants.

The classification codes and datasets utilized in this manuscript can be found in this link (accessed on 13 December 2024): https://github.com/LLLLIIIII/N-dealkylation-Classification. Our model demonstrated that using a weighted vote enhanced the accuracy of the basic vote classification across all models, which is consistent with findings from other prediction models in environmental research [38,39]. The model can expedite the selection of specific substrates from a vast array of amine pollutants for analytical validation, toxicity testing, and biological monitoring. In terms of screening efficiency, results for all compounds in the dataset can be obtained in less than two minutes using a standard computer. In contrast, traditional metabolic experiments and quantum chemistry calculations face several limitations, including cumbersome processes, high resource consumption, and low efficiency. For example, quantum chemistry calculations can take many hours to optimize the reaction transition states, intermediates, and products for each amine in the test set. Additionally, this requires the experimenter to possess a strong background in chemistry and significant prior experience. Furthermore, metabolic experiments necessitate considerable resources, including large quantities of microsomes and chemical reagents for product identification, which typically rely on expensive high-resolution mass spectrometry [40,41].

One of the key limitations of the current approach is that it does not take into account competing metabolic pathways and multiple metabolic transformations. In future work, it will be crucial to collect a more diverse range of data on amines and to explore the incorporation of other machine learning and artificial intelligence techniques, such as deep learning algorithms. These approaches may enhance the predictive capability for N-dealkylation reactions.

## 5. Conclusions

In this research, N-dealkylation reactions for 286 emerging amine pollutants with varying chemical structures were gathered and processed. A series of binary classification models were developed using molecular descriptors related to reactivity and structural fit to characterize the metabolic pathway. The models employed various algorithms, including random forest, gradient boosting decision tree, extreme gradient boosting, and multi-layer perceptron. The best-performing model, a gradient boosting decision tree classifier, achieved an accuracy of 81.0%, a sensitivity of 96.0%, and a MCC of 0.66. Additionally, experimental results indicated that a soft-voting ensemble model produced even better results, with an accuracy of 86.2%, sensitivity of 96.0%, and an MCC of 0.74. This ensemble model demonstrated robust and improved imputation accuracy across all base classification models, showcasing strong predictive performance. Furthermore, the performance of the ensemble model was comparable to other studies, highlighting the advantages of combining multiple machine learning algorithms while minimizing their individual weaknesses. This approach presents a valuable tool for predicting metabolic routes for emerging pollutants in environmental hazard assessments.

Future efforts should focus on identifying more aspects of metabolic routes to improve our method.

## Figures and Tables

**Table 1:** The molecular descriptors employed in classification models with their range, mean value, and type.

Name	Range	Mean Value	Type
ATSC1i	−42.10–11.98	−9.82	Autocorrelation
AATSC2v	−12.74–24.65	3.39	Autocorrelation
ETA_eta_B	−0.01–2.33	0.28	ExtendedTopochemicalAtom
ETA_dBeta	−18.00–4.50	−2.47	ExtendedTopochemicalAtom
CIC3	0.00–2.65	0.83	InformationContent
SMR_VSA6	0.00–99.36	19.09	MoeType
SlogP_VSA2	5.90–260.06	33.18	MoeType

**Table 2:** Optimized hyperparameters for RF, GBDT, XGB, and MLP classification models.

Machine Learning Algorithm	Hyperparameter
RF	n_estimators = 4, max_depth = 3
GBDT	n_estimators = 5, max_depth = 7
XGB	min_child_weight = 0.125, subsample = 0.8, colsample_bytree = 0.9, learning_rate = 0.01, n_estimators = 200
MLP	hidden_layer_sizes = (80, 100, 50), learning_rate_init = 0.01, max_iter = 800

**Table 3:** Performance metrics of single models in training set.

Single Classifier	Accuracy	Sensitivity	Specificity	Precision	F1 Score	MCC
RF	88.6%	93.1%	83.7%	87.7%	0.90	0.76
GBDT	87.3%	98.5%	72.4	82.6%	0.89	0.75
XGB	91.7%	99.2%	81.6	90.0%	0.93	0.83
MLP	86.4%	88.5%	83.7	87.8%	0.88	0.72

**Table 4:** Performance metrics of single models in testing set.

Single Classifier	Accuracy	Sensitivity	Specificity	Precision	F1 Score	MCC
RF	77.6%	84.0%	72.7%	70.0%	0.76	0.56
GBDT	81.0%	96.0%	69.7%	70.6%	0.81	0.66
XGB	79.3%	100.0%	63.6%	67.6%	0.80	0.65
MLP	79.3%	88.0%	72.7%	71.0%	0.78	0.60

**Table 5:** Performance metrics of consensus models in training set.

Consensus Classifier	Accuracy	Sensitivity	Specificity	Precision	F1 Score	MCC
RF+XGB+GBDT	90.4%	99.2%	78.6%	86.0%	0.92	0.86
RF+XGB+MLP	89.5%	93.8%	83.7%	88.4%	0.91	0.78
RF+GBDT+MLP	89.5%	93.8%	83.7%	88.4%	0.91	0.78
XGB+MLP+GBDT	89.5%	96.2%	80.6%	86.8%	0.91	0.78

**Table 6:** Performance metrics of consensus models in testing set.

Consensus Classifier	Accuracy	Sensitivity	Specificity	Precision	F1 Score	MCC
RF+XGB+GBDT	86.2%	96.0%	78.8%	77.4%	0.85	0.74
RF+XGB+MLP	84.5%	96.0%	75.8%	75.0%	0.84	0.71
RF+GBDT+MLP	84.5%	96.0%	75.8%	75.0%	0.84	0.71
XGB+MLP+GBDT	82.8%	96.0%	72.7%	72.7%	0.82	0.68

## Data Availability

The data presented in this study are available on request from the corresponding author.
